# Supplementary material for: Sketching and sampling approaches for fast and accurate long read classification
Source: BMC Bioinformatics. 2022 Oct 31;23:452. doi: 10.1186/s12859-022-05014-0 (PMC9624007; doi:10.1186/s12859-022-05014-0)
Supplement: Supplementary file 1 — Additional file 1﻿. Additional details and results related to the experiments in this manuscript. [file 12859_2022_5014_MOESM1_ESM.docx]

## **Supplementary Materials: Sketching and sampling approaches for fast and accurate long read classification**

Arun Das^*1^, Michael C. Schatz^1^

^1^Department of Computer Science, Johns Hopkins University, Baltimore, MD, 21218, USA

Contact: [arun.das@jhu.edu](mailto:arun.das@jhu.edu), [mschatz@cs.jhu.edu](mailto:mschatz@cs.jhu.edu)

**Supplementary Note 1. Versions of tools used**

| **Software** | **Version** |
| --- | --- |
| minimap2 | 2.22-r1101 |
| Kraken2 | 2.0.7-beta |
| centrifuge | 1.0.3-beta |
| CLARK | 1.2.6.1 |
| Winnowmap | 2.03 |
| MashMap | 2.0 |


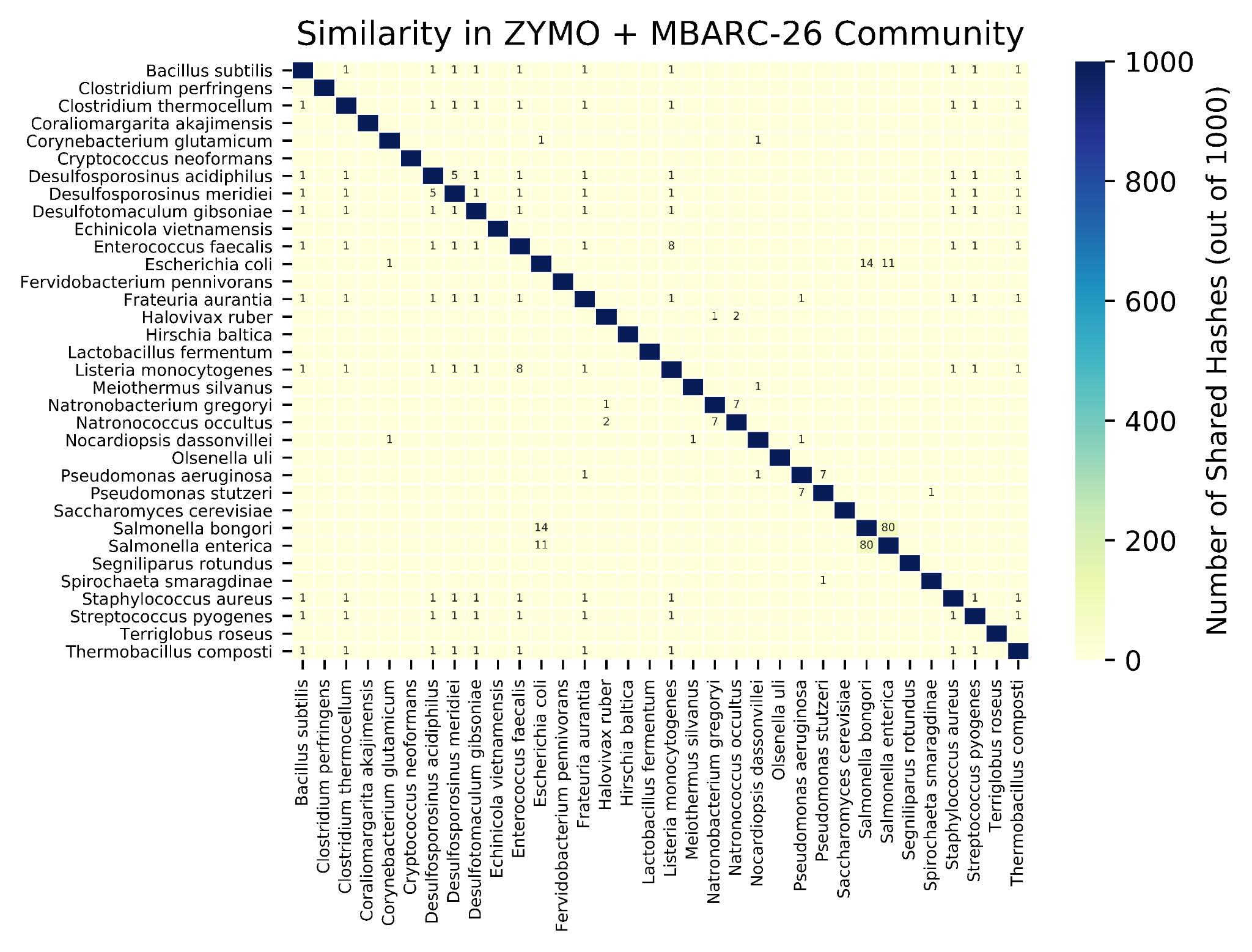


**Supplementary Figure 1: Similarity in the ZYMO + MBARC-26 Community:** Using Mash, we measured the similarity between each of the 34 members of the community. Each genome is sketched down to 1000 hashes, and these 1000 hash sketches are compared. All pairs of genomes that share at least one hash are shown on the plot above, with all genomes sharing 1000 hashes with themselves (as seen on the diagonal). The two most similar genomes are two strains of Salmonella, with 8% similarity, but only two other pairs have similarity over 1%. The low level of similarity between members of this community means classification is a much easier task.


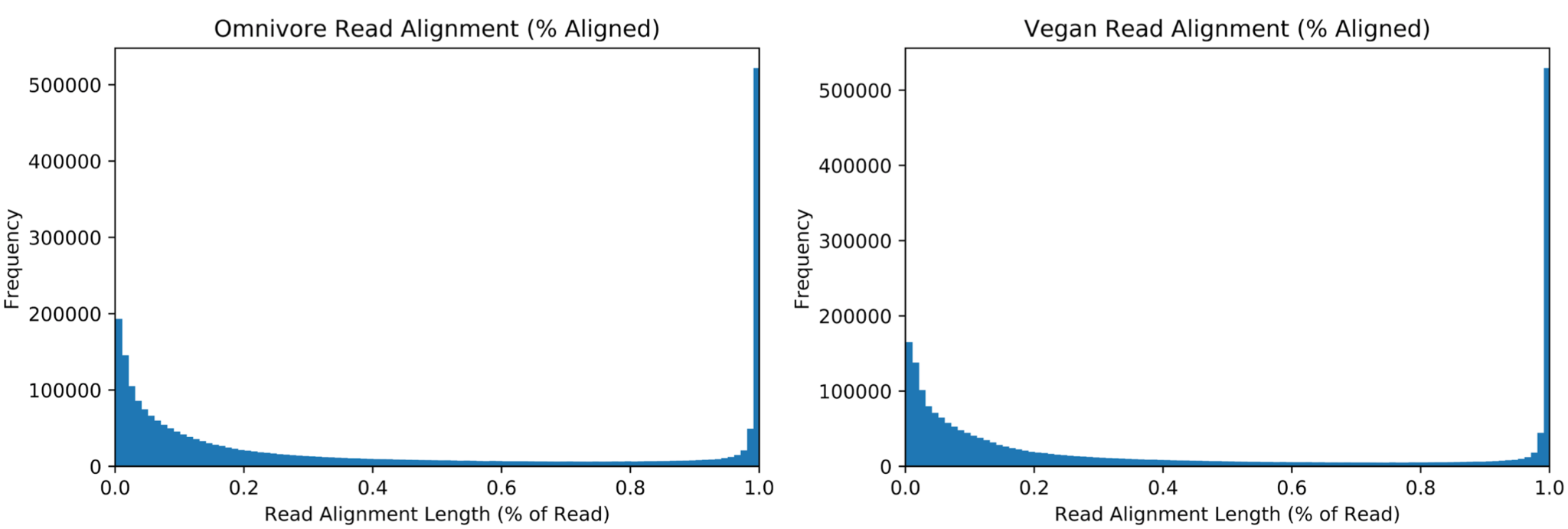


**Supplementary Figure 2: Minimap2 alignment lengths, as a percentage of read length, in two genuine metagenomics sequencing datasets.** Distribution of Minimap2 read alignments for genuine PacBio HiFi reads from an omnivore (left) and a vegan (right) to the CGR dataset. We observe a bimodal distribution, with the vast majority of reads either having only small sections aligned (<20%), or being almost completely aligned (>95%), and only a few reads falling in the middle.

**
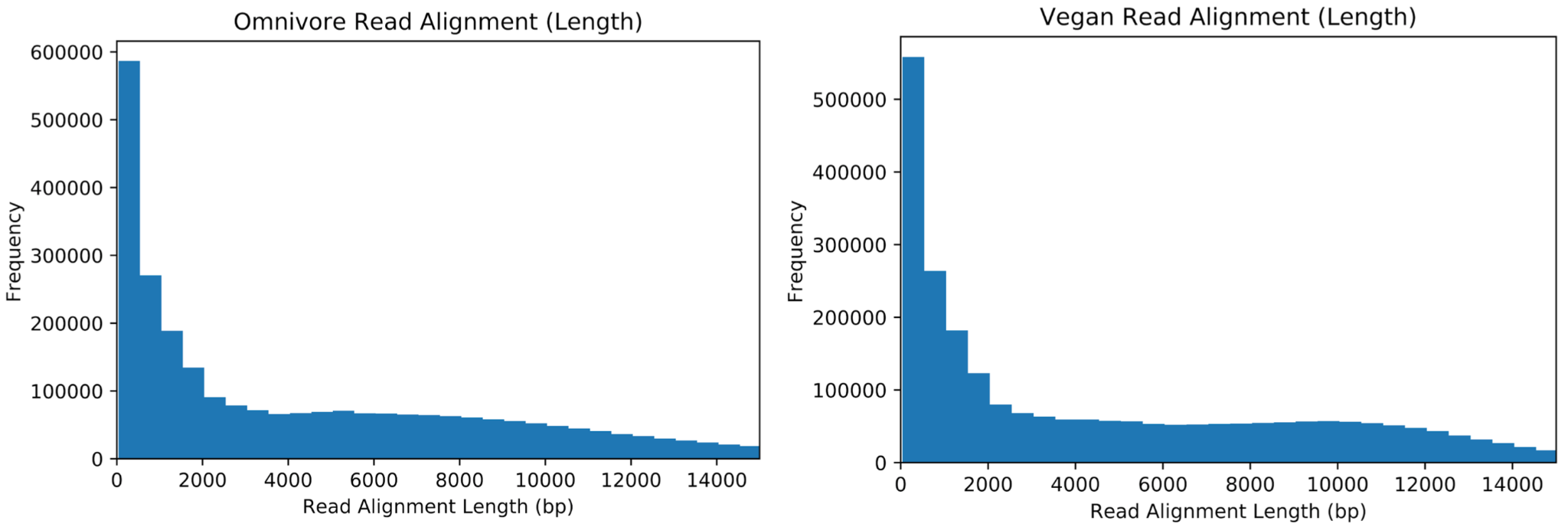
**

**Supplementary Figure 3: Minimap2 alignment lengths in two genuine metagenomics sequencing datasets.** Distribution of Minimap2 read alignments for genuine PacBio HiFi reads from an omnivore (left) and a vegan (right) to the CGR dataset. Unlike **Supplementary Figure 2**, using alignment lengths instead of the fraction of the read aligned allows varying read lengths to skew our analysis. Longer reads with a small fraction aligned may have longer alignments than fully aligned, shorter reads, despite the latter being classified with more certainty.

|  | **Read Lengths (Kb)** | | | | | | |  |
| --- | --- | --- | --- | --- | --- | --- | --- | --- |
| **Read Error (%)** |  | **0.5** | **2.5** | **5.0** | **10.0** | **25.0** | **50.0** | **MinHash** |
|  | **0.0** | 57.1 | 65.0 | 69.0 | 72.8 | 77.5 | 80.8 |  |
|  | **1.0** | 56.6 | 64.6 | 68.9 | 72.8 | 77.6 | 80.8 |  |
|  | **2.5** | 55.1 | 64.1 | 68.6 | 72.7 | 77.8 | 80.8 |  |
|  | **5.0** | 54.5 | 62.7 | 67.8 | 72.4 | 77.7 | 80.7 |  |
|  | **10.0** | 52.5 | 62.0 | 66.9 | 70.8 | 77.2 | 80.6 |  |
| **Read Error (%)** | **0.0** | 60.1 | 70.0 | 74.0 | 77.4 | 82.1 | 85.8 | **Minimizer** |
|  | **1.0** | 58.7 | 69.2 | 72.7 | 75.6 | 79.8 | 83.3 |  |
|  | **2.5** | 57.8 | 69.0 | 72.1 | 74.2 | 78.0 | 81.2 |  |
|  | **5.0** | 57.1 | 68.9 | 72.3 | 74.0 | 76.7 | 79.8 |  |
|  | **10.0** | 55.6 | 67.4 | 71.9 | 73.6 | 78.6 | 80.1 |  |
| **Read Error (%)** | **0.0** | 55.3 | 63.9 | 68.1 | 71.1 | 74.7 | 77.9 | **Uniform** |
|  | **1.0** | 54.8 | 63.5 | 67.6 | 70.9 | 73.7 | 75.9 |  |
|  | **2.5** | 53.3 | 63.1 | 67.1 | 70.5 | 72.9 | 74.8 |  |
|  | **5.0** | 52.8 | 62.4 | 66.4 | 69.5 | 72.6 | 73.7 |  |
|  | **10.0** | 52.1 | 61.9 | 66.0 | 68.9 | 72.1 | 72.2 |  |

**Supplementary Table 1: Microbial Classification Accuracy.** Classification accuracy across a range of read lengths and error rates, when using the three main sketching and sampling approaches (MinHash, Minimizer and Uniform).

| **# Target Matches** | **Microbial Classification Accuracy** | | | | |
| --- | --- | --- | --- | --- | --- |
|  | **MinHash** | **WMH (W = 2)** | **WMH (W = 5)** | **Minimizer** | **Uniform** |
| **1** | 17.1 | 17.2 | 17.5 | 16.9 | 18.9 |
| **2** | 21.0 | 21.5 | 22.1 | 20.5 | 24.7 |
| **3** | 25.4 | 25.9 | 26.2 | 25.3 | 29.9 |
| **4** | 34.9 | 35.4 | 35.9 | 35.1 | 33.1 |
| **5** | 38.6 | 40.6 | 41.0 | 40.6 | 37.5 |
| **10** | 48.9 | 51.0 | 51.8 | 49.2 | 42.0 |
| **15** | 56.7 | 57.3 | 57.6 | 58.1 | 51.8 |
| **20** | 60.3 | 60.8 | 61.2 | 61.7 | 56.4 |
| **25** | 62.7 | 63.1 | 63.4 | 64.3 | 59.4 |
| **30** | 64.6 | 65.2 | 65.4 | 66.2 | 62.8 |
| **50** | 68.8 | 69.6 | 70.1 | 70.7 | 66.1 |
| **75** | 71.3 | 71.9 | 72.4 | 73.7 | 68.3 |
| **100** | 72.8 | 73.6 | 74.0 | 75.6 | 70.9 |
| **150** | 75.7 | 76.4 | 76.8 | 78.0 | 72.8 |
| **200** | 77.8 | 78.2 | 78.4 | 79.6 | 74.5 |

**Supplementary Table 2: Effect of sketch size on microbial classification accuracy.** Classification accuracy on the CGR dataset using sketching and sampling approaches, across a range of target matches. We observe steady decreases in genome-level classification accuracy as the number of target matches decreases.

|  | **% Human (H) and Contaminant (C) Reads Identified** | | | | | | | | | |
| --- | --- | --- | --- | --- | --- | --- | --- | --- | --- | --- |
|  | **MinHash** | | **WMH (W = 2)** | | **WMH (W = 5)** | | **Minimizer** | | **Uniform** | |
| **# Target Matches** | **H** | **C** | **H** | **C** | **H** | **C** | **H** | **C** | **H** | **C** |
|  |  |  |  |  |  |  |  |  |  |  |
| **1** | 45.5 | 42.1 | 46.0 | 42.2 | 46.3 | 42.5 | 46.1 | 43.1 | 45.6 | 42.7 |
| **2** | 59.4 | 56.3 | 60.4 | 56.7 | 60.8 | 57.0 | 63.2 | 60.8 | 61.0 | 60.5 |
| **3** | 72.6 | 70.9 | 73.1 | 71.4 | 73.4 | 71.6 | 75.0 | 73.7 | 72.4 | 70.4 |
| **4** | 83.2 | 82.4 | 83.8 | 82.6 | 84.1 | 82.8 | 84.5 | 83.1 | 82.6 | 80.3 |
| **5** | 89.4 | 88.5 | 90.5 | 88.9 | 90.8 | 89.0 | 90.8 | 89.0 | 88.6 | 88.1 |
| **10** | 96.1 | 95.2 | 96.4 | 95.3 | 96.4 | 95.3 | 96.6 | 95.3 | 95.4 | 94.9 |
| **15** | 97.5 | 96.7 | 97.6 | 96.8 | 97.7 | 96.9 | 97.8 | 96.9 | 97.0 | 96.5 |
| **20** | 98.5 | 97.5 | 98.5 | 97.7 | 98.5 | 97.7 | 98.7 | 97.8 | 98.1 | 97.1 |
| **25** | 98.6 | 98.1 | 98.6 | 98.2 | 98.6 | 98.2 | 98.9 | 98.3 | 98.4 | 98.0 |
| **30** | 98.6 | 98.5 | 98.6 | 98.5 | 98.7 | 98.5 | 98.9 | 98.5 | 98.5 | 98.2 |
| **50** | 98.9 | 98.8 | 98.9 | 98.8 | 99.0 | 98.8 | 99.0 | 98.9 | 98.8 | 98.4 |
| **75** | 99.1 | 99.0 | 99.1 | 99.0 | 99.2 | 99.0 | 99.3 | 99.0 | 99.0 | 98.9 |
| **100** | 99.3 | 99.3 | 99.3 | 99.3 | 99.3 | 99.3 | 99.4 | 99.3 | 99.1 | 99.1 |
| **150** | 99.5 | 99.4 | 99.5 | 99.4 | 99.5 | 99.4 | 99.5 | 99.4 | 99.5 | 99.3 |
| **200** | 99.5 | 99.4 | 99.5 | 99.4 | 99.5 | 99.5 | 99.5 | 99.5 | 99.5 | 99.3 |

**Supplementary Table 3: Effect of sketch size on contaminant detection.** Percentage of human (H) or “contaminant” microbial (C) reads correctly identified as being of interest or from contaminants respectively, across a range of sketching and sampling approaches. We find that accuracy remains relatively constant until the number of target matches, and therefore the sketch size, drops below 0.2-0.3% of the original k-mers.

|  | **MH** | **WMH w/ Multiplier** | | | | | **OMH** |
| --- | --- | --- | --- | --- | --- | --- | --- |
|  |  | **1** | **2** | **5** | **10** | **15** |  |
| **Accuracy (%)** | 72.2 | 72.7 | 72.7 | 72.8 | 72.8 | 72.8 | 72.4 |

**Supplementary Table 4: Effect of weight and order on MinHash accuracy.** The inclusion of weight sees slight increases in performance over unweighted MinHash, while the addition of order results in smaller improvements.

| **Approach** | **Number of k-mers stored** | **# Lookups performed during Classification** |
| --- | --- | --- |
| Exhaustive | 1,600,728,9970 | ~48,000,000,000 |
| MinHash | 60,382,132 | 604,985,465 |
| Minimizer | 69,382,660 | 687,003,545 |
| Uniform | 61,098,861 | 608,955023 |

**Supplementary Table 5: Overhead of proposed sketching and sampling approaches.** We count the number of 21-mers stored in the screen and the number of comparisons performed while classifying simulated 10Kb, 1% error, 10x coverage reads from the CGR microbial community, which has 4.85GB of sequence across 1,310 organisms. The screens are designed to capture 100 target matches per read. The sketching and sampling approaches only use a fraction of the unique k-mers present in the data, and thus have significantly reduced overhead compared to an exhaustive approach that stores all unique k-mers in the source genomes and looks up every k-mer present in the read sets. Note that the number of lookups performed in the exhaustive approach is an estimate, as the exhaustive approach is impractical to run in practice.

| **Approach** | **Size on Disk (GB)** |
| --- | --- |
| Kraken2 (Standard) | 58.0 |
| Kraken2 (Custom) | 60.2 |
| Centrifuge (Standard) | 7.9 |
| Centrifuge (Custom) | 8.2 |
| CLARK (Standard) | 94.0 |

**Supplementary Table 6: Comparison of the uncompressed database sizes for the three index-based approaches.** All three “Standard” databases are available directly from the developers of the tools. The pre-prepared Kraken2 and Centrifuge indexes cover all bacteria, archaea, viral and human genomes in Refseq, and the CLARK index is a complete set of bacterial genomes. The “custom” databases are further augmented to explicitly include the sequences in the CGR dataset, by adding the sequences and their taxonomy IDs to the existing database. This augmentation does not seem to add a significant amount of sequence.

| **Approach** | **Runtime (minutes)** |
| --- | --- |
| Minimap2 | 4680.3 |
| Kraken2 | 120.2 |
| Centrifuge | 410.7 |

**Supplementary Table 7: Runtime of alignment- and index-based approaches.** Time taken (computed as the “real” time from Unix’s “time” command) to classify simulated reads in our contaminant detection experiments, using 10Kb, 1% error, 10x coverage reads drawn from the CGR microbial dataset and the human reference genome GRCh38 (totalling ~7.85 Gb of sequence). This does not include the index-generation time for Kraken2 or Centrifuge, whose indexes were downloaded from the developers. All approaches are run with 32 threads. From these results, the speedup of index-based approaches over Minimap2 is clear, and as Minimap2 is already significantly faster than full sequence-to-sequence alignment, the limitations of alignment-based methods for large datasets should be evident. Within the two index-based approaches, Kraken2 is considerably faster, though it does require a significantly larger database (**Supplementary Table 6**).
